# The Information–Motivation–Behavioral Skills Model Revisited: A Network-Perspective Structural Equation Model Within a Public Sexually Transmitted Infection Clinic Sample of Hazardous Alcohol Users

**DOI:** 10.1007/s10461-016-1446-2

**Published:** 2016-06-03

**Authors:** Steven A. John, Jennifer L. Walsh, Lance S. Weinhardt

**Affiliations:** 1 Joseph J. Zilber School of Public Health, University of Wisconsin-Milwaukee, P.O. Box 413, Milwaukee, WI 53201-0413 USA; 2 Department of Psychiatry and Behavioral Medicine, Center for AIDS Intervention Research (CAIR), Medical College of Wisconsin, Milwaukee, WI USA; 3 Department of Psychiatry and Human Behavior, Alpert Medical School, Brown University, Providence, RI USA; 4 Department of Behavioral and Social Sciences, School of Public Health, Brown University, Providence, RI USA; 5 Center for Behavioral and Preventive Medicine, The Miriam Hospital, Providence, RI USA

**Keywords:** IMB model, Behavioral theory, Sexual risk behavior, HIV/AIDS, Hazardous alcohol users

## Abstract

The information–motivation–behavioral skills (IMB) model is useful for understanding sexual risk behavior, but has not been tested with hazardously-drinking sexually transmitted infection (STI) clinic patients, a subpopulation at greater HIV risk, or with a network-perspective sexual risk behavior outcome. Participants (*N* = 569) were STI clinic patients who screened positive for hazardous drinking and risky sexual behavior. Sexual risk behavior (SRB) was operationalized as a latent variable with three indicators: (1) number of sexual partners, (2) number of unprotected sex occasions with primary partner, and (3) number of unprotected sex occasions with non-primary partner(s). Preliminary analyses suggested SRB was best operationalized as a latent variable with two indicators, while unprotected sex with primary partners should be considered separately. In structural models with good fit, the IMB model was generally supported. The IMB model functioned differently for non-primary and multiple partners compared to primary partners in STI clinic patients with hazardous alcohol use.

## Introduction

Sexually transmitted infections (STIs) remain highly prevalent within many communities in the United States, and disparities in STIs between Black or African-Americans and Whites keep prevention of STIs a national priority [1, 2]. STI clinic patients, in particular, are in need of additional attention given their inherent elevated risk for infection and repeat infection [3–5]. Individuals with repeated or untreated infections are at greater risk for medical complications including infertility, ectopic pregnancy, and pelvic inflammatory disease [6]. Patients that continually acquire STIs are also at risk for contracting HIV through continued engagement in sexual risk behavior, and additional evidence suggests an epidemiological synergy with STIs that increases risk for HIV by two- to five-fold regardless of symptomology [7–9].

STI clinic patients classified as hazardous alcohol users represent a subpopulation of STI clinic patients at greater risk for HIV. Physiologically, alcohol use reduces immune function, which makes the likelihood of seroconversion higher upon exposure to HIV [10]. Behaviorally, the causal pathway between alcohol use and sexual risk behavior is less clear. Higher risk of HIV and other STIs in the context of alcohol use could be the result of behavioral disinhibition, decreased condom-use skills, or attitudes during sex after alcohol consumption, but proposed mediating third variables (e.g., personality traits and disorders) further muddle causal interpretation [10, 11]. Although research into the causal mechanisms explaining the association between alcohol use and STI risk is still ongoing, alcohol use is associated with increased sexual risk-taking among STI clinic patients [e.g., 12], making interventions for this population of special interest.

Given the limited resources in many STI clinic settings, offering intensive interventions targeting both alcohol risk reduction and sexual risk in addition to providing STI counseling and testing services may not be feasible. For this reason, it is important to identify the key predictors of HIV and STI risk behavior for alcohol-using clinic patients. Testing theory-based models of risk behavior specifically within this sub-population of STI clinic patients may provide direction for researchers and public health practitioners alike. The prevalence of recent alcohol use has been reported as high as 81 % within a large, urban public health STI clinic in the US, with 17 % of those reporting participation within alcohol treatment previously [13]. These findings suggest that alcohol-using STI clinic patients could represent a noteworthy proportion of total patients within some public STI clinics in the US. These patients may be in need of different intervention strategies compared to patients who do not engage in hazardous alcohol use. A more nuanced understanding of how health behavior theories operate for particular high-risk populations can aid in the adaptation or development of population-specific behavioral interventions to be experimentally tested or evaluated within clinical practice settings.

**Fig. 1:**
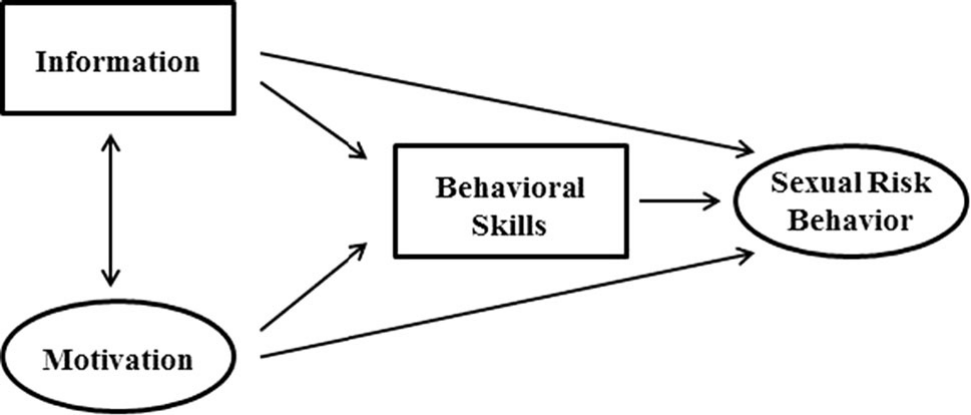
Information–motivation–behavioral skills conceptual model. *Note* information–motivation–behavioral skills model adapted from Fisher et al. [56]

Theory-based research is common in the area of STI and HIV prevention, and many prevention interventions have been based on the information–motivation–behavioral skills (IMB) model [14, 15]. Fisher and Fisher [14] proposed and tested the IMB model based on a critique of previous research, and they argued that risk reduction interventions were most impactful when based on a conceptual framework; population specific; and focused on information, motivation, and behavioral skills. Now frequently used, the IMB model posits that individuals must be informed, motivated, and behaviorally skilled to initiate and maintain HIV prevention behavior. Specifically, individuals must have information that is relevant to the transmission and prevention of STIs and easy to apply in their social setting. Motivation to engage in risk reduction and HIV prevention activities must be supported by individual attitudes and perceived social norms, and highly motivated and informed individuals must have the skills to perform the HIV prevention activity, including self-efficacy, to effectively reduce their risk for HIV and other STIs (see Fig. 1 for conceptual model) [14, 16].

IMB model research is specifically needed with alcohol-using STI clinic patients because theoretical predictors of sexual risk behavior may operate differently with this unique high-risk population. The IMB model has not been widely used in predicting sexual risk behavior for alcohol users; rather, IMB model antecedents that view intentions as the largest determinant of health behavior [e.g., Theory of Planned Behavior, 17] have been used extensively. For example, researchers find robust evidence in support of an association between alcohol consumption and higher intentions to engage in unprotected sex, which is moderated by heightened sexual arousal, in meta-analytic review [18]. Despite the similarities in theoretical constructs (e.g., attitudes, subjective norms, and intentions) between the IMB model and its antecedents, the IMB model differs in that it does not posit intentions as the largest or most proximal predictor of sexual risk behavior. Intentions are instead a component of the motivational construct of the IMB model that is hypothesized to operate through behavioral skills before behavioral action is taken. Therefore, we believe it is necessary to test the IMB model with this high-risk sample of alcohol users to determine the role of other theoretical constructs—mainly behavioral skills—in mediating the association between intentions and other motivations and sexual risk behavior.

Since conceptualization, the IMB model for HIV prevention has been widely tested using structural equation modeling (SEM), which allows for the use of latent constructs to reduce the influence of measurement error along with the simultaneous consideration of associations between multiple constructs [19, 20]. However, the measurement of risk is a weakness of prior tests of the IMB model. First, the majority of previous research has included only a single measure of condom use consistency—the percentage of sexual events involving condom use—as an outcome [21–32]. A key limitation of using percentage of condom use is the inability to differentiate levels of risk for individuals who report the same percentage of condom use but have different frequencies of sexual behavior or different types of sexual partners. Second, measuring consistency of condom use without considering number of sexual partners may also lead to an incomplete conceptualization of risk that does not account for a more complex understanding of the proximal sexual network.

Previous research has suggested that predictors of unprotected sex may differ for events involving primary versus non-primary partners. For example, Senn et al. [30] found that partner dependence, operationalized as perceived safety, economic, and emotional dependence on a partner, significantly predicted more frequent unprotected sex and a higher proportion of condomless sex with steady (i.e., primary) partners. This study found no significant associations between partner dependence and unprotected sex frequency or proportion with non-steady partners, suggesting the necessity of separately considering sexual risk events with primary and non-primary partners. Additionally, results from prior research have sometimes differed based on whether percentage of condom use or number of unprotected sexual acts is considered as an outcome. For example, Mittal et al. [26] found different results between number and percentage of condom use, particularly between motivation and condom use, across both total sexual events and those specific to steady partners. Although no “gold standard” of measurement has been determined [33], the public health perspective on measurement suggests that each specific unprotected sex event increases risk of STI transmission [34]. A broader conceptualization of risk behavior includes both number of sexual partners and number of unprotected sex events, aligning with a sexual network perspective that places individuals at higher risk for an STI with each additional partner. As such, we use outcome frequency measures to account for proximal sexual network size and each sexual risk event, a conceptual priority within our sexual network perspective.

The current study aims to model the IMB model using a sexual network perspective latent variable. Viewed from a network perspective, sexual risk may differ based on the number of sexual partners, number of unprotected sexual occasions with a primary partner, and number of unprotected sexual occasions with non-primary partner(s). We are not the first to modify the dependent variable in testing the IMB model to account for additional sexual risk measurement. Mustanski et al. [35] modeled a composite indicator of sexual risk for minority youth seeking health services. Within this risk indicator, number of sexual partners and consistency in condom use were measured. Bazargan et al. [36] also modeled a latent dependent variable measuring: (1) ever having sex without condoms, (2) number of sexual partners, and (3) age at first sexual intercourse. Nonetheless, both of these indicators were limited when attempting to account for each specific sexual risk event, a conceptual priority within our sexual network perspective.

Given elevated sexual risk-taking and HIV risk among STI clinic patients who are hazardous alcohol users and the need to identify key intervention targets for this population, we tested the IMB model in a sample attending a Midwestern public STI clinic for confidential HIV counseling and testing. The purpose of this research is not intended to add further evidence to the relationship between alcohol use and sexual risk behavior, but rather to test a theory-based model of risk behavior within a unique population—STI clinic patients with a history of hazardous alcohol use—and perspective using a latent outcome variable with multiple measures of risk. In line with the IMB model, we hypothesized that higher HIV prevention information and motivation would predict higher HIV prevention behavioral skills; higher behavioral skills, in addition to higher information and motivation, would then predict lower sexual risk behavior. Thus, behavioral skills would partially mediate the relationships between information and risk behavior and between motivation and risk behavior. Although we hypothesized direct pathways between all constructs and sexual risk behavior as initially conceptualized by Fisher and Fisher [14], we acknowledged that information may not have a direct association with risk behavior given mixed results from previous IMB models [37]. Key innovations of the current study included the unique, high-risk sample and the multidimensional conceptualization of sexual risk behavior.

## Methods

### Participants

Participants were recruited from a large, Midwestern public STI clinic as part of enrollment into a randomized controlled trial. Research staff determined if individuals were eligible for study recruitment if the participants: (1) were 18 years of age or older; (2) self-reported unprotected vaginal or anal intercourse with two or more sexual partners, an anonymous partner, or an injection drug using partner in the past 3 months, or had been diagnosed and treated for an STI other than HIV in the past 3 months; (3) scored 8 or higher on the AUDIT screening tool for hazardous alcohol use [38, 39]; (4) agreed to a confidential HIV test when offered during standard STI clinical practice; and (5) had no HIV-positive test result in the past.

Of the 1150 patients screened eligible, 606 participants consented to enroll in the study and *N* = 569 had complete data used for analysis. Participants had a mean age of 34.41 (SD = 10.69). Seventy percent of participants were male, 89 % Black or African American, and less than 5 % Hispanic. Eighty-six percent of participants had a high school diploma, high school equivalent, or less education, and less than 14 % classified as a full- or part-time student. Most participants were unemployed (i.e., 75 %), and 87 % made less than $1000 per month in income. Seventy-three percent of participants were single and never married, and 94 % classified their sexual orientation as heterosexual.

### Measurement

Survey assessments were completed using Audio Computer-Assisted Self-Administered Interviewing (ACASI) software. Scales were used to measure IMB model constructs. Information and behavioral skills were measured as individual indicators with a single scale each, and motivation was measured by scales of condom social norms, condom attitudes, and condom intentions. Dependent variables within our model included count data of sexual partners and unprotected sexual occasions.

#### Information

The HIV-KQ-18 [40] was used to measure HIV knowledge, which we operationalized as information. This scale has been validated previously with good internal consistency, test–retest reliability, and valid comparison measures to a longer version HIV knowledge questionnaire, and we found similarly good internal consistency (see Table 1). Participants answered 18 questions with *true, false*, and *don’t know* responses. Answers were then recoded as correct or incorrect, with all “don’t know” responses coded as incorrect. An example question from this scale includes “coughing and sneezing DO NOT spread HIV” [40].

**Table 1:** Descriptive statistics and reliabilities of model indicators (*N* = 569)

Indicator	Mean	SD	Range	α^a^
HIV-KQ-18	13.00	3.58	0–18	0.80
Social norms	22.26	7.79	6–36	0.82
Attitudes	35.40	6.82	10–60	0.58
Intentions	33.33	7.31	7–42	0.75
Self-efficacy	59.41	18.81	0–80	0.91
Number of sexual partners	4.55 (1.26)^b^	14.10 (0.79)^b^	0–300 (0–5.71)^b^	–
Number of unprotected sex occasions with primary partner	25.46 (2.21)^b^	52.34 (1.56)^b^	0–1000 (0–6.91)^b^	–
Number of unprotected sex occasions with non-primary partner(s)	5.20 (0.98)^b^	12.62 (1.12)^b^	0–99 (0–4.61)^b^	–

^a^Reliability (Cronbach’s alpha)

^b^log(x + 1) transformed in parentheses

*SD* standard deviation

#### Motivation

The IMB model construct motivation was measured using three indicator scales of condom social norms, condom attitudes, and condom intentions. Social norms were measured using a 6-item survey with 6-point semantic differential response categories from *strongly disagree* to *strongly agree.* An example question includes “current sexual partners think we should use condoms every time.” A 10-item scale was used to measure attitudes, and similar response categories of *strongly disagree* to *strongly agree.* One example question from this scale is “the use of condoms can make sex more stimulating.” Lastly, intentions were measured using a 7-item scale with *strongly disagree* to *strongly agree* response categories, and an example question includes “the next time I have sex, I will do only safe sex.” Similar indicators for motivation have been used previously within IMB model research [14, 16, 41].

#### Behavioral Skills

HIV prevention self-efficacy, a National Institute of Mental Health Multisite HIV Prevention Trial measure [42], was used to measure behavioral skills within our model. Eight items measured self-efficacy with responses of *not at all confident* to *completely confident* scored from 0 to 10. Gender-specific situational questions were used, and an example question is “how confident are you that you could bring up the issue of condoms or safe sex in a conversation in this situation?” [42]. Self-efficacy has been frequently used as a proxy for behavioral skills within the IMB model literature [22–25, 27, 28, 30, 32, 35, 36, 43–49].

#### Sexual Risk Behavior

We operationalized our outcome latent variable of sexual risk behavior using three indicators of behavior within the last 90 days: (1) number of sexual partners, (2) number of unprotected sex occasions with primary partner, and (3) number of unprotected sex occasions with non-primary partner(s). The first indicator measured number of sexual partners separately by gender [“how many different men (women) have you had sex with in the past 90 days?”], and these were combined to form a single indicator. The second and third indicators were created by combining questions measuring vaginal and anal sex occasions separately [“how many times have you had unprotected vaginal (anal) sex with your primary partner in the past 90 days?”; “how many times have you had unprotected vaginal (anal) sex with others in the past 90 days?”].

### Statistical Analysis

Data were prepared using STATA 13.1 (Intercooled), and we analyzed our conceptual model using the operationalized latent variable of sexual risk behavior in Lisrel version 9.1 (Student) using a maximum likelihood estimator [50]. Descriptive statistics and intra-class coefficients using Cronbach’s alpha are presented in Table 1 and the variances, covariances, and correlations of indicator variables are presented in Table 2. IMB model indicators were standardized [(x_i_ − x_mean_)/SD] to reduce multicollinearity, and outcome variables log(x + 1) transformed to improve normality. Information and behavioral skills had single indicators as scales, thus we set the error variance to [(1 − intra-class coefficient) * sample variance]. Motivation is estimated using the three indicator variables, as is sexual risk behavior. Motivation and information are allowed to covary. We include direct paths from information and motivation to sexual risk behavior as well as indirect paths from these constructs to behavior through behavioral skills as conceptualized in the IMB model by Fisher and Fisher [14]. Model fit was determined using multiple, established fit indices. Specifically, we used the *χ* ^2^ badness-of-fit index, root mean square error approximation (RMSEA), non-normed fit index (NNFI), and comparative fit index (CFI) to guide an estimation of overall model fit. We assumed good model fit when the *χ* ^2^/*df* ratio was 3 or less, RMSEA ≤ 0.05, NNFI > 0.95, and CFI > 0.95, and nested models were considered significantly different when the *χ* ^2^ difference test resulted in a *p* value ≤ 0.05 [20, 50–52].

**Table 2:** Variances, covariances, and correlations of indicator variables

	1	2	3	4	5	6	7	8
1. HIV-KQ-18	0.985	−*0.072*	−*0.097*	−*0.026*	*0.133*	−*0.048*	*0.127*	−*0.076*
2. Subjective Norms	−0.071	1.000	*0.247*	*0.453*	*0.335*	−*0.083*	−*0.277*	−*0.231*
3. Attitudes	−0.096	0.247	1.000	*0.241*	*0.088*	*0.001*	−*0.052*	−*0.037*
4. Intentions	−0.025	0.453	0.241	1.000	*0.294*	−*0.105*	−*0.196*	−*0.170*
5. Self-efficacy	0.132	0.335	0.088	0.294	1.000	−*0.266*	−*0.013*	−*0.300*
6. Sexual partners	−0.038	−0.066	0.001	−0.083	−0.211	0.629	*0.113*	*0.590*
7. Unprotected sex with primary partners	0.197	−0.432	−0.081	−0.306	−0.020	0.140	2.434	*0.263*
8. Unprotected sex with non-primary partners	−0.085	−0.258	−0.041	−0.191	−0.336	0.524	0.460	1.255

Covariances in lower left, variances along diagonal, and correlations in upper right italicized; covariances and variances were standardized for variables 1–5, and variable 1 is not equal to one due to rounding

## Results

### Sexual Risk Behavior: Preliminary Model

**Fig. 2:**
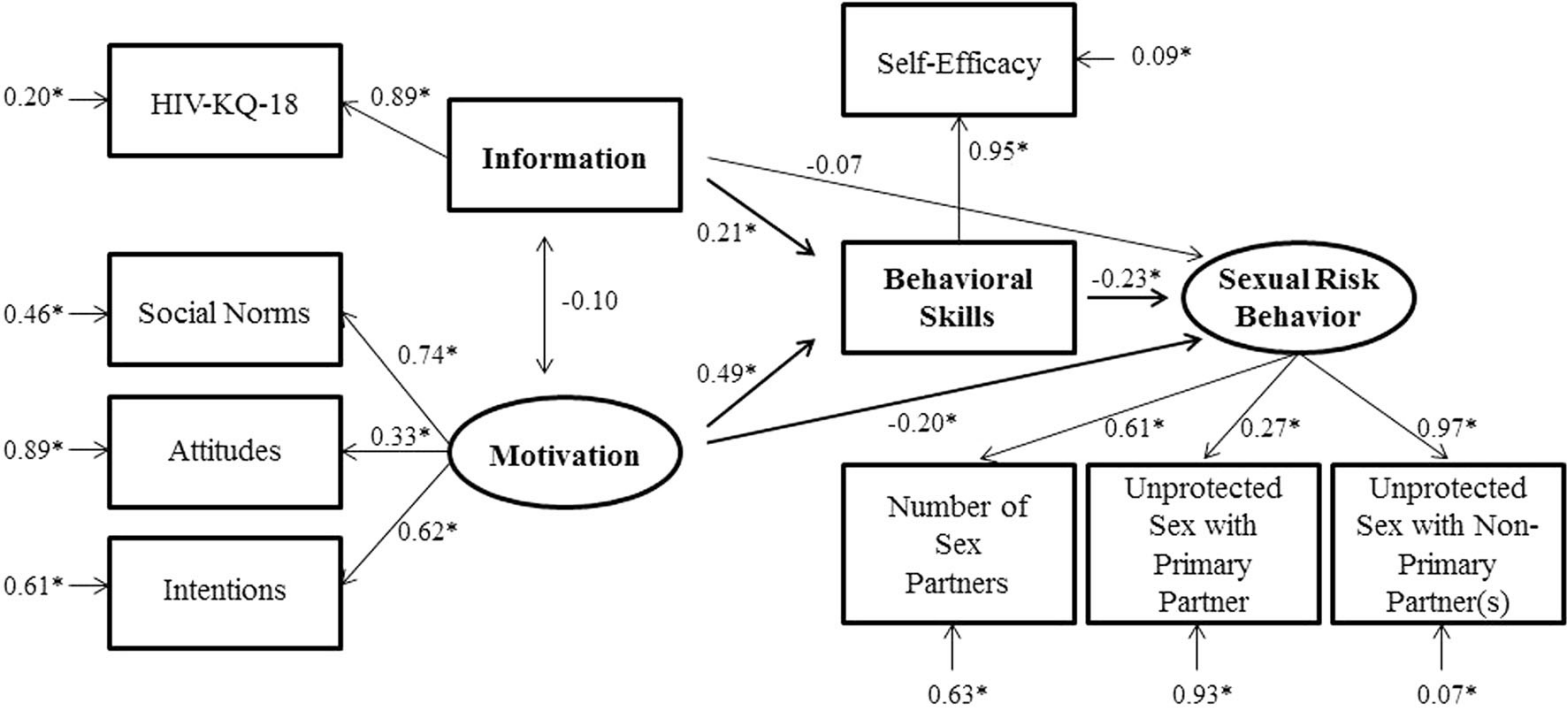
Preliminary model path diagram completely standardized of the information–motivation–behavioral skills model predicting general sexual risk behavior among STI clinic patients with hazardous alcohol use (*N* = 569). *Notes* * *p* < 0.05; all disturbance terms were significant (*p* < 0.05), but removed for interpretation ease; this model had adequate but less than ideal fit [χ^2^(16) = 80.98, *p* < 0.01; RMSEA = 0.085 (0.067–0.103 90 % CI); NNFI = 0.852; CFI = 0.915]

We originally conceptualized sexual risk behavior as a latent construct with three indicators: (1) number of sexual partners, (2) number of unprotected sex occasions with primary partner, and (3) number of unprotected sex occasions with non-primary partner(s). However, a preliminary model with adequate but less than ideal fit [*χ* ^2^(16) = 80.98, *p* < 0.01; RMSEA = 0.085 (0.067–0.103 90 % CI); NNFI = 0.852; CFI = 0.915] had a low factor loading for unprotected sex with primary partners (see Fig. 2), suggesting the construct of sexual risk behavior was not unidimensional. We therefore included unprotected sex with primary partners as a separate outcome variable. Although we anticipated the ability to successfully model sexual risk behavior from a sexual network perspective, our results provided evidence to suggest that a single construct of sexual risk behavior cannot be modeled as a unidimensional measure of risk within this sample. We postulated the low factor loading for primary partners and model misfit to be the result of potential differences in behavioral scripts between primary and non-primary partners. Individuals with multiple partners, and thus non-primary sexual partners, may engage in different behavior and negotiate condom use differently based on an appraisal of risk or relationship closeness [30, 53]. Therefore, we respecified this model of sexual risk behavior into two separate models of risk: (1) sexual risk behavior with outside partners, and (2) sexual risk behavior with primary partners.

### Final Models

**Fig. 3:**
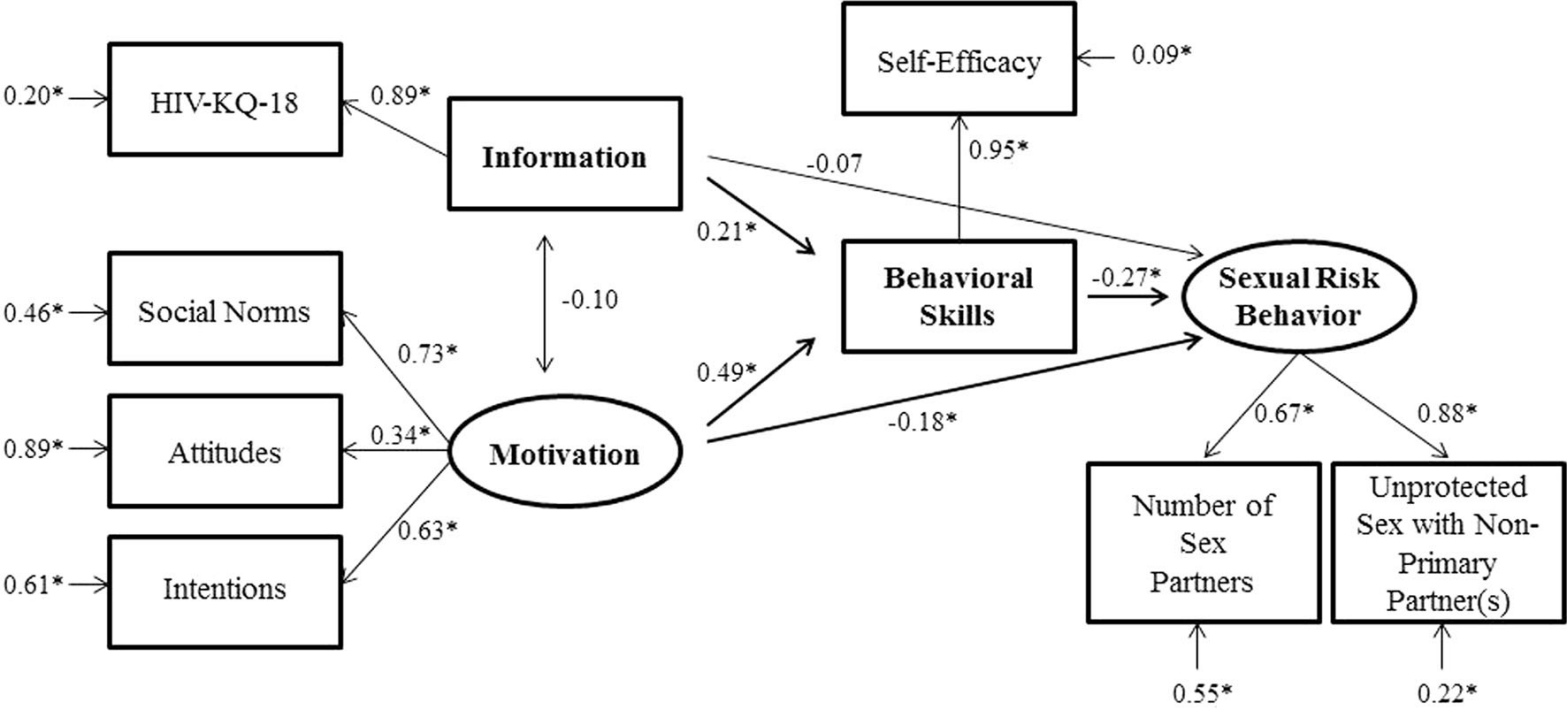
Path diagram completely standardized of the information–motivation–behavioral skills model predicting sexual risk behavior with outside partners among STI clinic patients with hazardous alcohol use (*N* = 569). *Notes* * *p* < 0.05; all disturbance terms were significant (*p* < 0.05), but removed for interpretation ease; this model had acceptable fit [χ^2^(10) = 21.42, *p* = 0.02, χ^2^/*df* ratio = 2.14; RMSEA = 0.045 (0.018–0.071 90 % CI); NNFI = 0.963; CFI = 0.982]

**Fig. 4:**
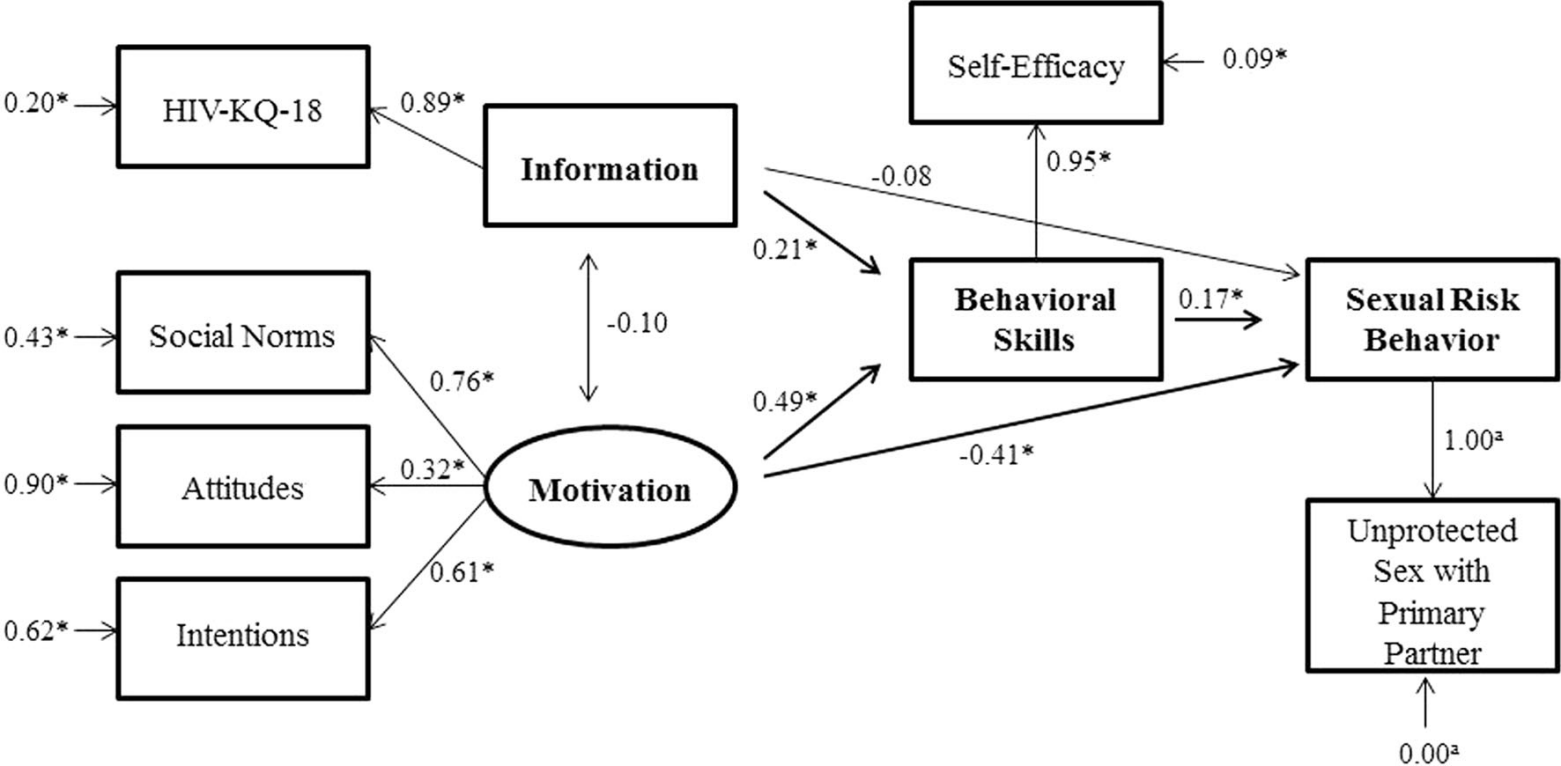
Path diagram completely standardized of the information–motivation–behavioral skills model predicting sexual risk behavior with primary partners among STI clinic patients with hazardous alcohol use (*N* = 569). *Notes* * *p* < 0.05; ^a^ single indicator with no error adjustment; all disturbance terms were significant (*p* < 0.05), but removed for interpretation ease; this model had acceptable fit [χ^2^(6) = 12.50, *p* = 0.05, *χ* ^2^/*df* ratio = 2.08; RMSEA = 0.044 (0.000–0.078 90 % CI); NNFI = 0.957; CFI = 0.983]

After respecification, the final models retained had good model fit. Specifically, the model of sexual risk behavior with outside partners had acceptable model fit [χ^2^(10) = 21.42, *p* = 0.02, *χ* ^2^/*df* ratio = 2.14; RMSEA = 0.045 (0.018–0.071 90 % CI); NNFI = 0.963; CFI = 0.982] and had significantly better fit than our preliminary model [*χ*_diff_^2^(6) = 59.56, *p* < 0.001]. The path diagram of this first respecified model is illustrated in Fig. 3. Higher behavioral skills significantly predicted less sexual risk behavior (β = −0.27, *p* < 0.01). Behavioral skills fully mediated the association of information with sexual risk behavior (β_indirect_ = 0.21, *p* < 0.01), and partially mediated the association of motivation with sexual risk behavior (β_direct_ = −0.18, *p* < 0.05; β_indirect_ = 0.49, *p* < 0.01). Because we were also interested in sexual risk behavior with primary partners, we tested an un-nested comparison model of unprotected sex with primary partners excluding number of sexual partners and unprotected sex occasions with non-primary partners. The un-nested comparison model had similar acceptable fit [χ^2^(6) = 12.50, *p* = 0.05, χ^2^/*df* ratio = 2.08; RMSEA = 0.044 (0.000–0.078 90 % CI); NNFI = 0.957; CFI = 0.983], but the structural model of the IMB constructs changed dramatically. Higher behavioral skills predicted more unprotected sexual occasions with the primary partner (β = 0.17, *p* < 0.01). Similar to our first final model, behavioral skills fully mediated the association of information with sexual risk behavior (β_indirect_ = 0.21, *p* < 0.01), and partially mediated the association of motivation with sexual risk behavior (β_direct_ = −0.41, *p* < 0.01; β_indirect_ = 0.49, *p* < 0.01). This second final model also had significantly better fit than the preliminary model in a nested comparison [*χ*_diff_^2^(10) = 68.48, *p* < 0.001]. The full path diagram of this second final model is illustrated in Fig. 4, and the standardized beta coefficients, standard errors, and Z-scores for estimates from all models are presented in Table 3.

**Table 3:** Standardized beta coefficient estimates, standard errors, and z-scores from structural equation models

Parameters	Estimate	SE	*z*-score
Preliminary model			
Information ↔ motivation	−0.102	0.038	−1.765
Information → behavioral skills	0.206	0.050	4.416**
Motivation → behavioral skills	0.490	0.082	7.768**
Information → sexual risk behavior	−0.070	0.027	−1.428
Motivation → sexual risk behavior	−0.199	0.045	−2.890**
Behavioral skills → sexual risk behavior	−0.225	0.031	−3.667**
Disturbance for information	1.000	0.058	13.506**
Disturbance for motivation	1.000	0.078	7.006**
Disturbance for behavioral skills	0.738	0.053	12.673**
Disturbance for sexual risk behavior	0.860	0.032	6.248**
Sexual risk behavior with outside partners			
Information ↔ motivation	−0.102	0.038	−1.759
Information → behavioral skills	0.206	0.050	4.414**
Motivation → behavioral skills	0.491	0.082	7.761**
Information → sexual risk behavior	−0.069	0.031	−1.314
Motivation → sexual risk behavior	−0.177	0.052	−2.478*
Behavioral skills → sexual risk behavior	−0.274	0.037	−4.137**
Disturbance for information	1.000	0.058	13.506**
Disturbance for motivation	1.000	0.078	6.935**
Disturbance for behavioral skills	0.737	0.053	12.658**
Disturbance for sexual risk behavior	0.840	0.038	6.176**
Sexual risk behavior with primary partners			
Information ↔ motivation	−0.102	0.038	−1.770
Information → behavioral skills	0.206	0.050	4.414**
Motivation → behavioral skills	0.487	0.077	7.949**
Information → sexual risk behavior	0.075	0.084	1.555
Motivation → sexual risk behavior	−0.412	0.145	−5.870**
Behavioral skills → sexual risk behavior	0.166	0.090	3.010**
Disturbance for information	1.000	0.058	13.506**
Disturbance for motivation	1.000	0.076	7.536**
Disturbance for behavioral skills	0.741	0.053	12.762**
Disturbance for sexual risk behavior	0.851	0.136	15.194**

*SE* standard error

* *p* < 0.05; ** *p* < 0.01

## Discussion

Our preliminary model of sexual risk behavior using a sexual network perspective resulted in a structural equation model with less than ideal fit. This preliminary analysis modeled a sexual risk behavior latent variable combining three indicators of behavior within the previous 90 days: (1) number of sexual partners, (2) number of unprotected sex occasions with primary partner, and (3) number of unprotected sex occasions with non-primary partner(s). This lack of acceptable model fit was not the result of the IMB model, rather our hypothesized conceptualization of risk. Model fit significantly improved when we separated unprotected sex with primary partners from the other two sexual risk behavior indicators, suggesting low correlation between primary partner risk and the other outcome variables. Another contributing factor to modest model fit within our preliminary model could be the result of a difference in how behavioral skills operated within the IMB model between non-primary and multiple partners compared to primary partners. These findings suggest the IMB model may predict behavior differently for non-primary and multiple partners as compared to primary partners for this high-risk population. Specifically, we found that behavioral skills had the expected negative correlation with risk behavior with non-primary and multiple partners, but a positive association with the number of unprotected sexual acts with main partners. This finding is consistent with some research that suggests different predictors of sexual risk for primary and non-primary partners [30]. Bazargan et al. [22] found that perceiving a monogamous relationship with a partner to be predictive of higher behavioral skills, but a decrease in condom use; behavioral skills may not be protective when looking at unprotected sex with main partners. While we identify some potential congruence of our findings with prior research, additional investigation is needed with alcohol-using STI clinic patients to better understand the association between behavioral skills and unprotected sex with main partners. Specifically, research considering potential moderators of the association between behavioral skills and sexual risk behavior is called for.

One potential moderator of special relevance to the current population is alcohol consumption within sexual encounters. Behavioral disinhibition from alcohol use could be stronger in sexual encounters with primary partners compared to non-primary partners, moderating the effect between HIV prevention self-efficacy and unprotected sex worthy of additional investigation. The inhibitory cues of higher self-efficacy could be stronger for sexual encounters with non-primary partners regardless of alcohol use, but perceptions of higher self-efficacy could be misinterpreted as confidence in a low-risk unprotected sex event with their primary partner potentially caused by alcohol-related behavioral disinhibition. Kiene et al. [54] applied the alcohol myopia theory [55] to study the moderating factors between condom use self-efficacy and unprotected sex with event-level data to find that alcohol consumption before sex disrupted the inhibitory cues of stronger self-efficacy. Based on our own findings, we suggest future research to determine whether this moderating effect differs based on partnership type.

Our results add to existing literature which found inconsistent results regarding the role of information in the IMB model. Specifically, past studies found that information does not always have a direct effect on sexual risk behavior, but many studies suggest that information remains a necessary component of HIV prevention interventions because of the influence knowledge has on behavioral skills. Our results fit with other studies that found that information significantly predicted behavioral skills, but did not directly predict sexual risk behavior [21, 22, 24, 32, 44, 48, 49, 56]; however, our results conflict with those that found no effect of information [25–28, 31, 35] and those with a direct relationship with condom use [22, 43]. It has been argued that the importance of HIV prevention information may be attenuated within populations with higher levels of knowledge [37], and we found low levels of HIV prevention knowledge within this sample of alcohol-using STI clinic patients providing additional evidence in support of this hypothesis.

The effects of motivation and behavioral skills within our models also provide evidence consistent with many IMB studies, but conflict with others. Our results suggest motivation had a direct effect on behavioral skills, but also had a direct effect on sexual risk behavior. This partial mediation effect of motivation on sexual risk behavior through behavioral skills is consistent with other IMB model research [21, 25, 27–29, 31, 35, 44, 56], but conflicts with evidence of a fully-mediating effect [32, 36, 43, 49]. Thus, motivation and behavioral skills remain important components of the IMB model, but some populations may rely more heavily upon behavioral skills to enact protective behavior compared to others. Our model adds to existing literature suggesting that the IMB model is to be tested within specific populations before planning intervention activities [14, 16].

This theory-based research with alcohol-using STI clinic patients may aid researchers and practitioners in adapting and developing further intervention strategies to help this vulnerable population reduce their risk for subsequent STIs including HIV. Although prior research has provided ample support of the IMB model, no previous studies have tested the model with this specific high-risk population. This research allowed us to identify a discrepant finding from other high-risk groups—mainly a difference in how behavioral skills operated based on partnership type. This suggests that interventions targeting self-efficacy for HIV prevention behaviors for patients with primary partners may not be adequate to reduce unprotected sexual behaviors. Instead, additional emphasis should be placed on knowledge, motivation, and potential factors moderating the association between behavioral skills and unprotected sex. In summary, this research prompts additional research into the moderating effects of sexual partnership type between IMB model factors and sexual risk behavior, particularly related to HIV prevention self-efficacy.

The results of this analysis should be interpreted with caution given a number of limitations. First, the cross-sectional nature of this study limits our ability to substantiate any causal effects or rule out any equivalent models, but the findings of our study help support existing evidence published to date. Second, our data are reliant on accurate self-reporting of sensitive behaviors. Although we used ACASI survey methodology to increase the accuracy of our data, we cannot ignore potential response bias. Lastly, the use of self-efficacy as a proxy for behavioral skills could have impacted how behavioral skills operated within the tested IMB model. Although the use of self-efficacy is common within IMB model literature, our finding that HIV-prevention self-efficacy predicted more unprotected sex with primary partners potentially limits our immediate intervention planning abilities to reduce sexual behavior with primary partners using the IMB model. In addition to more focused efforts on knowledge and motivations, intervention strategies based on different theoretical models should also be considered to address risk behavior with primary partners.

Despite these limitations, this research has several noteworthy strengths. First, we were the first to empirically test the IMB model with a multidimensional conceptualization of sexual risk that includes both number of partners and counts of unprotected sexual acts with different partner types. Our strategy allowed us to account for each sexual risk event and sexual partner through frequency measures, a conceptual priority within our sexual network perspective. Although we were unable to identify a unidimensional measure of risk with this specific high-risk population, we recommend additional research with other populations because of the potential public health implications of using a sexual network perspective—mainly a latent observation of risk that incorporates the number of sexual partners and each unprotected sex event. Second, we were the first to empirically test the IMB model within hazardous alcohol users seeking HIV counseling and testing, a specific subpopulation of STI clinic patients with noteworthy risk. Specifically, we found that the IMB model was supported within this sample of alcohol-using STI clinic patients. Moreover, the theoretical components of our IMB models match previous research conducted in this STI clinic that found success in reducing STIs at 1-year follow-up assessments through the use of a full IMB model intervention, as compared to deconstructed intervention components, for risk reduction counseling [13].

## Conclusions

In this study, we found empirical support for the IMB model using a multidimensional conceptualization of sexual risk behavior among hazardous alcohol users seeking HIV counseling and testing within a Midwestern public STI clinic. Our findings suggest the IMB model functions differently for non-primary and multiple partners compared to primary partners in STI clinic patients with hazardous alcohol use. Intervention strategies should incorporate these findings into planning interventions for STI clinic patients engaging in hazardous alcohol use to reduce their risk for subsequent STIs including HIV. Alternative theoretical models, including adaptations of the IMB model and exploration of moderating factors, should also be tested to address risk behavior with primary partners for this specific high-risk population. Our research adds evidence in support of the IMB model as a theory-based model that predicts sexual risk behavior, but additional research is needed to more fully understand the implications of the findings related to sexual risk behavior with primary partners.
